# Lymphoid Stress Surveillance Response Contributes to Vitiligo Pathogenesis

**DOI:** 10.3389/fimmu.2018.02707

**Published:** 2018-11-20

**Authors:** Liisi Raam, Epp Kaleviste, Marina Šunina, Helen Vaher, Mario Saare, Ele Prans, Maire Pihlap, Kristi Abram, Maire Karelson, Pärt Peterson, Ana Rebane, Kai Kisand, Külli Kingo

**Affiliations:** ^1^Department of Dermatology, University of Tartu, Tartu, Estonia; ^2^Dermatology Clinic, Tartu University Hospital, Tartu, Estonia; ^3^Institute of Biomedicine and Translational Medicine, University of Tartu, Tartu, Estonia

**Keywords:** vitiligo, interferons, MICA/MICB, WIPI1, LC3, *EOMES*, B cells, autophagy

## Abstract

Vitiligo is a chronic multifactorial depigmentation disorder characterized by the destruction and functional loss of melanocytes. Although a direct cytotoxic T cell attack is thought to be responsible for melanocyte damage, the events leading to the loss of self-tolerance toward melanocytic antigens are not understood. This research aimed to identify novel cellular and molecular factors that participate in vitiligo pathogenesis through the application of gene expression and immunofluorescence analysis of skin biopsy samples along with immunophenotyping of circulating cells. Our study provides insights into the mechanisms involved in melanocyte destruction. The upregulation of stress-ligand MICA/MICB, recognized by activating receptors on innate and innate-like T cells, imply involvement of lymphoid stress surveillance responses in vitiligo lesions. A simultaneous increase in the expression of transcription factor *EOMES* that is characteristic for innate-like virtual memory T cells, suggest a similar scenario. Local lymphoid stress surveillance has been previously associated with the amplification of systemic humoral responses that were mirrored in our study by increased T follicular helper cells and switched memory B cell proportions in patients with active vitiligo. In addition, microtubule-associated protein light chain 3 staining was compatible with the activation of autophagy in keratinocytes and in the remaining melanocytes of vitiligo lesional skin.

## Introduction

Vitiligo is an acquired multifactorial skin pigment disorder manifesting mainly as white macules on the skin due to melanocyte destruction. It is the most common depigmentation disorder affecting 0.5–1% of the population worldwide (1). Although depigmentation rarely causes any physical symptoms, vitiligo may cause psychosocial problems and impair quality of life (2). In addition, several comorbidities including autoimmune, systemic, and dermatologic diseases have been described (3).

Multiple mechanisms are supposed to be involved in melanocyte disappearance including genetic predisposition, environmental triggers, metabolic abnormalities, impaired renewal of melanocytes, and altered inflammatory and immune responses (4). Susceptibility to vitiligo is determined by several gene loci, most of which encode immunoregulatory factors and proteins involved in melanocyte function (5). As elevated levels of reactive oxygen species have been observed in vitiligo lesional skin and in melanocytes isolated from the skin of vitiligo patients (6), it is believed that in genetically susceptible individuals, environmental factors such as ultraviolet radiation, chemical agents, or mechanical trauma may lead to uncontrolled production of reactive oxygen species in the skin and consequently activate autoimmune processes resulting in melanocyte destruction (4). In addition to melanocyte dysfunction, alterations in keratinocytes also play a role. Keratinocytes in depigmented epidermis are more vulnerable to apoptosis and produce lesser amounts of melanogenic mediators than in normal skin. Furthermore, expression of genes involved in keratinocyte differentiation and cornification is dysregulated in lesional epidermis (7, 8).

Increased oxidative stress and alterations in both melanocytes and keratinocytes can induce production of pro-inflammatory cytokines and signals important for the activation of pathogenic immune cells in vitiligo patients (4, 9). It is thought that activation of the innate immune system through pathogen recognition receptors is impaired, which then induces autoimmunity in genetically predisposed patients (4). Melanocyte-specific antibodies, which are uncommon in healthy persons, have been found to circulate in the blood and deposit in the skin of vitiligo patients (10). CD8+ and CD4+ T cells are consistently found at the edge of actively depigmenting skin (11). Moreover, circulating skin-homing melanocyte-specific cytotoxic T lymphocytes have been found in the blood of vitiligo patients (12–15). However, the initial events that breach self-tolerance to melanocyte-specific antigens still remain unidentified.

This study aimed to identify the novel cellular and molecular patterns that determine vitiligo pathogenesis. Our results are consistent with the involvement of IFNs (16), and point to the activation of lymphoid stress surveillance responses and autophagy in diseased skin, but also strongly suggest the participation of germinal center reaction during the active phase of vitiligo.

## Materials and methods

### Study sample

A case-control study was conducted in Dermatology Clinic, Tartu University Hospital. Ethical approval was obtained from the Research Ethics Committee of the University of Tartu. All the participants signed a written informed consent. We recruited 21 patients with non-segmental vitiligo (6 males, 15 females; age range 19–60 years) and 28 control subjects (8 males, 20 females; age range 24–57 years) for the study. Vitiligo patients were recruited from the outpatient department of the Dermatology Clinic, Tartu University Hospital. The diagnosis of vitiligo based on the loss of pigmentation with typical localization and depigmented macules on the skin under Wood's lamp. Seven of the patients had active and 14 had stable vitiligo. Active vitiligo was defined as a condition in which development of new lesions or extension of old lesions was revealed in 3 months before examination. None of the patients had received any specific treatment for at least a month prior to the study. Control subjects were recruited from among health care personnel, medical students, and patients who turned to the dermatologic outpatient clinic for excision of naevi. They were free from chronic dermatoses and a positive family history of vitiligo. All of the participants were unrelated individuals of Caucasian race living in Estonia. For all the participants, form on the socio-demographic and clinical data was filled. Characteristics of the participants are shown in Table S1.

One skin punch biopsy sample (3–4 mm in diameter) from non-sun-exposed skin was taken from all control subjects. Two skin punch biopsy samples (3–4 mm in diameter) were collected from 16 vitiligo patients, one from the marginal zone of involved skin (hereinafter lesional skin) and another from non-sun-exposed uninvolved skin (hereinafter non-lesional skin). The marginal zone of involved skin was chosen for gene expression study as inflammatory cell infiltration locates predominantly in the area between lesional and non-lesional vitiligo skin (10). For immunofluorescence assay, skin from the center of the vitiligo lesion and non-lesional skin was biopsied from 3 patients with vitiligo and embedded in OTC formulation. All the skin samples were instantly frozen in liquid nitrogen and stored at −80°C until RNA extraction and immunofluorescence analysis.

From 20 vitiligo patients and 24 control subjects, 16 ml venous blood was collected into BD Vacutainer® CPT™ Cell Preparation Tubes with sodium heparin (BD Biosciences, Franklin Lakes, New Jersey, USA) and to separate plasma and peripheral blood mononuclear cells (PBMCs) from other blood cells the tubes were centrifuged at 1,500 g for 30 min. Plasma was collected and stored at −20°C. Isolated PBMCs were washed twice with phosphate-buffered saline (PBS) and were stored in freezing medium in liquid nitrogen for flow cytometry.

### RNA purification and qRT-PCR

Gene expression analysis was carried out on skin biopsy samples of 16 vitiligo patients. 24 control subjects were included in this analysis. A total RNA was isolated from the skin using RNeasy Fibrous Tissue Mini Kit (Qiagen, Valencia, California, USA) or miRNeasy Mini Kit (Qiagen, Valencia, California, USA) according to the manufacturer's instructions. For RNA extraction the skin biopsy samples were homogenized in 700 μl of the QIAzol Lysis Reagent (Qiagen, Valencia, California, USA) by a gentleMACS^TM^ Dissociator (Miltenyi Biotec, Heidelberg, Germany) using M tubes. The concentration and quality of the RNA were assessed with a NanoDrop ND-1000 spectrophotometer (Thermo Fisher Scientific, Wilmington, Massachusetts, USA). cDNA was synthesized from 5000 ng of total RNA using oligo-dT and SuperScript® III Reverse Transcriptase (Life Technologies, Carlsbad, California, USA) according to the manufacturer's instruction.

For amplification of the PCR product, SYBR® Green (Life Technologies, Carlsbad, California, USA) master mix was used. qRT-PCR analysis was carried out on ViiA^TM^ 7 Real-Time PCR system (Life Technologies, Carlsbad, California, USA). Primer sequences are listed in Table S2. The relative gene expression levels were calculated using the comparative Ct (ΔΔ*C*t) method and normalized to the expression of Actin, Beta (ACTB).

### Cytokine testing

The concentration of proteins TNF-α, IL-1b, IL-1Ra, IL-2, IL-5, IL-6, CXCL8, CXCL10, IFN-γ, Granulocyte–Colony Stimulating Factor (G-CSF) and Granulocyte-Macrophage-(GM)-CSF was measured in plasma of 18 patients with vitiligo and 24 control subjects. The xMAP technology on Luminex 200 (Luminex Corporation, Austin, Texas, USA) was used. The Milliplex MAP multiplex assay was conducted in a 96-well microplate format according to the manufacturer's instructions (Millipore, Billerica, Massachusetts, USA).

### Flow cytometry

Surface marker expression on PBMCs from 17 vitiligo patients and 18 control subjects was assessed by flow cytometry. Cells were stained in flow cytometry staining buffer (PBS with 0.5% bovine serum) for 20 min at 4°C with antibodies listed in Table S3. Stained cells were analyzed using LSRFortessa flow cytometer (BD Biosciences) and FACSDiva version 6 (BD Biosciences) software. The optical detector configuration is provided in Table S4.

### Cell isolation and culture

Pooled, normal human epidermal keratinocytes (Promocell, Heidelberg, Germany) were cultured in Keratinocyte-SFM medium with supplements (Life Technologies, Carlsbad, California, USA) at 37°C in 5% humidified CO_2_ incubator. For two-dimensional (2D) cultures, cells with density of 20,000 cells per well on 24-well plates were seeded and for 3D keratinocyte culture in air liquid interface, 50,000 cells per well were seeded on ThinCert Cell Culture Inserts (0.4 μm pore, 0.33 cm2) on 24-well plates (Greiner Bio-One, Kremsmünster, Austria). For the 3D culture, Keratinocyte-SFM medium with supplements and Dulbecco's Modified Eagle Medium (both from Life Technologies, Carlsbad, California, USA) containing High Glucose, GlutaMAX™ and Pyruvate in 1:1 ratio were used. For the 2D culture, Keratinocyte-SFM medium with supplements (Life Technologies, Carlsbad, Califonia, USA) was used. Melanocytes and fibroblasts were isolated and cultured as described by Reemann et al. (17).

Monocyte derived Langerhans cells (moLC) were generated as follows. PBMCs were isolated from buffy coat by Ficoll-Hypaque Plus (Amersham Biosciences, Piscataway, USA) density gradient centrifugation. Monocytes were isolated using MACS anti-CD14 beads (Miltenyi Biotec, Bergisch Gladbach, Germany) according to the manufacturer's protocol up to purity over 95%. The isolated cells were cultured in RPMI 1640 medium (supplemented with 10% fetal calf serum, 1% penicillin and streptomycin, all from PAA Laboratories, Pasching, Austria) at a density of 1–1.5 × 106 cells/ml. moLC were differentiated for seven days in the presence of GM-CSF (50 ng/ml), IL-4 (25 ng/ml), TGFβ (10 ng/ml), and TSLP (5 ng/ml), all from R&D Systems, Minneapolis, USA (18).

### Immunofluorescence

Immunofluorescence was performed on 5 μm thick frozen sections of skin biopsy samples. The samples were fixed with 4% formaldehyde and permeabilized with 0.2% Triton X-100 in PBS. After that, the slides were stained using the Alexa Fluor™ 594 Tyramide SuperBoost™ Kit and goat anti-mouse IgG (Thermo Fisher Scientific, Wilmington, Massachusetts, USA) according to the manufactures manual. Briefly, the slides were blocked 60 min at room temperature (RT) with 10% normal goat serum and then incubated with primary antibodies for 60 min at RT. The used antibodies were mouse monoclonal antibody to microtubule-associated protein light chain 3 (LC3) (nanoTools, Teningen, Germany) and mouse anti-human MICA/MICB antibody (BioLegend, San Diego, California, USA). After that the slides were incubated 60 min at RT with poly-HRP-conjugated goat anti-mouse secondary antibody. For the signal enhancement, the tyramide working solution was added for 6 min at RT and the stop solution was used to halt the HRP reaction. To identify melanocytes, the sections were incubated with anti-TYRP1 (tyrosinase related protein 1) rabbit polyclonal antibody (Atlas Antibodies, Sweeden) at 4°C overnight and incubated with Alexa Fluor® 488 conjugated Goat anti-Rabbit IgG (H+L) Secondary Antibody (1:1,000, ThermoFisher Scientific, 1:500) for 60 min at RT. After nuclear counterstain with DAPI (4′,6-Diamidine-2′-phenylindole dihydrochloride, 1 μg/mL) for 10 min the slides were washed three more times in PBS and covered with fluorescent mounting medium (Dako, Santa Clara, California, USA) and coverslips. Images were obtained with FV1200 confocal microscope (Olympus, Tokyo, Japan). For Figure **5E**, the fluorescence signal marking LC3 and DAPI were quantified using Fiji (19) with built-in options.

### Statistical analysis

Statistical analysis was performed using the R statistical software (https://www.r-project.org/). The mRNA expression values and the fluorescence signal ratios were log-transformed before the statistical testing to adhere with the assumptions of the normal distribution. The conformity to a normal distribution was assessed using the Kolmogorov–Smirnov test. Comparisons between vitiligo patients and control subjects were made using the unpaired Student's *t*-test. Comparing vitiligo lesional skin with non-lesional skin, the paired Student's *t*-test was used. In mRNA expression analysis and immunofluorescence signal quantification, if vitiligo lesional and non-lesional skin was compared to healthy control skin, the Dunnett's correction was applied to adjust for multiple comparisons. The mean and standard deviation of the log-transformed mRNA expression values were back-transformed to linear scale for plotting, which is shown as the geometric mean ×÷ geometric standard deviation on the graphs. Correlation was assessed with the Pearson's correlation coefficient. A *p*-value < 0.05 was considered significant.

## Results

### Lesional vitiligo skin is characterized by an interferon signature

**Figure 1:**
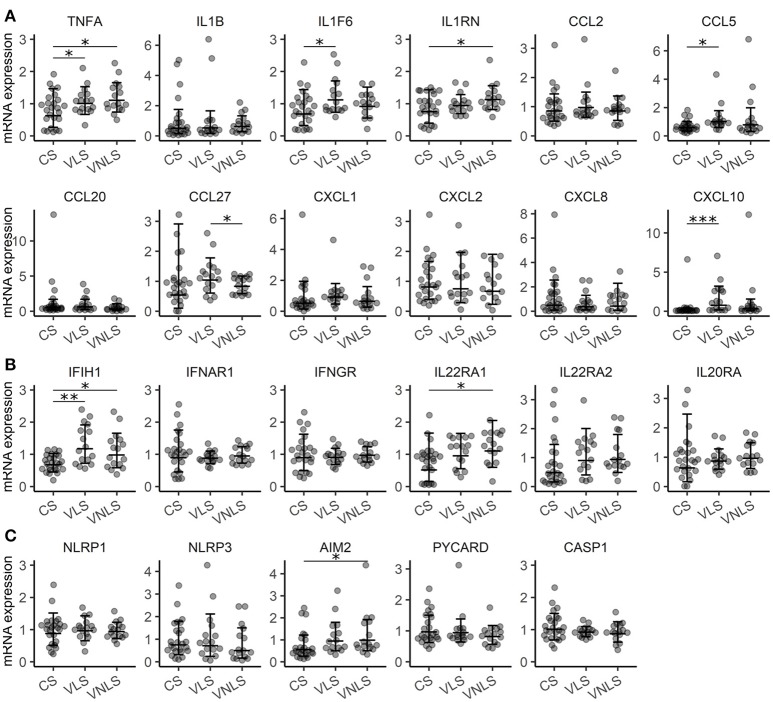
Gene expression signature is consistent with a very mild inflammatory response in vitiligo. Relative expression of mRNAs encoding **(A)** cytokines, **(B)** receptors and **(C)** components of the inflammasome in the skin of control subjects (CS), and in the lesional skin (VLS) and non-lesional skin (VNLS) of vitiligo patients. Geometric mean ×÷ geometric standard deviation is indicated. *p < 0.05; **p < 0.01; and ***p < 0.001.

Although vitiligo lesional skin does not manifest substantial macroscopic signs of inflammation, the processes that occur at the cellular and molecular level should leave some informative traces to local gene expression that would help dissect the processes leading to melanocyte destruction. Like multiple previous studies (7, 20–23) we could only detect very moderate upregulation of some inflammatory cytokines such as *TNF* (*p* < 0.05) and *IL36A* (alias IL1F6) (*p* < 0.05) in vitiligo lesional or non-lesional skin (Figure 1A). The transcripts of *IFNG* and Th17 specific cytokines remained undetectable in control as well as in vitiligo biopsy samples. In addition, IL-1 inhibitory *IL1RN* (*p* < 0.05) showed a slight increase in vitiligo non-lesional skin. From the studied chemokines, only *CCL5* (*p* < 0.05) and *CXCL10* (*p* < 0.001) were significantly upregulated in vitiligo lesional skin compared with healthy control skin (Figure 1A). *CXCL10* is an interferon (IFN) regulated gene like *IFIH1* (24), which was also upregulated in vitiligo lesional (*p* < 0.01) and in non-lesional skin (*p* < 0.05) (Figures 1A,B). From the studied cytokine receptors, only *IL22RA1* (*p* < 0.05) expression was increased in the non-lesional skin of vitiligo patients compared with the skin of control subjects (Figure 1B). Whether this implicates the IL-22 pathway in vitiligo pathogenesis remains unknown as *IL22* transcripts were below the detection limit. However, upregulation of *IL22* in the PBMCs of vitiligo patients has been reported earlier (25). From the studied inflammasome-related genes only *AIM2* was slightly elevated in vitiligo non-lesional skin (Figure 1C). As the vitiligo group consisted of patients with active as well as stable disease, it remained possible that the differences between groups could be masked by the high proportion of patients without an active process in their lesions. Therefore, we compared the gene expression between patients with active or stable disease; however, the data did not reveal higher inflammatory cytokine expression in active vitiligo skin (Table S5).

**Figure 2:**
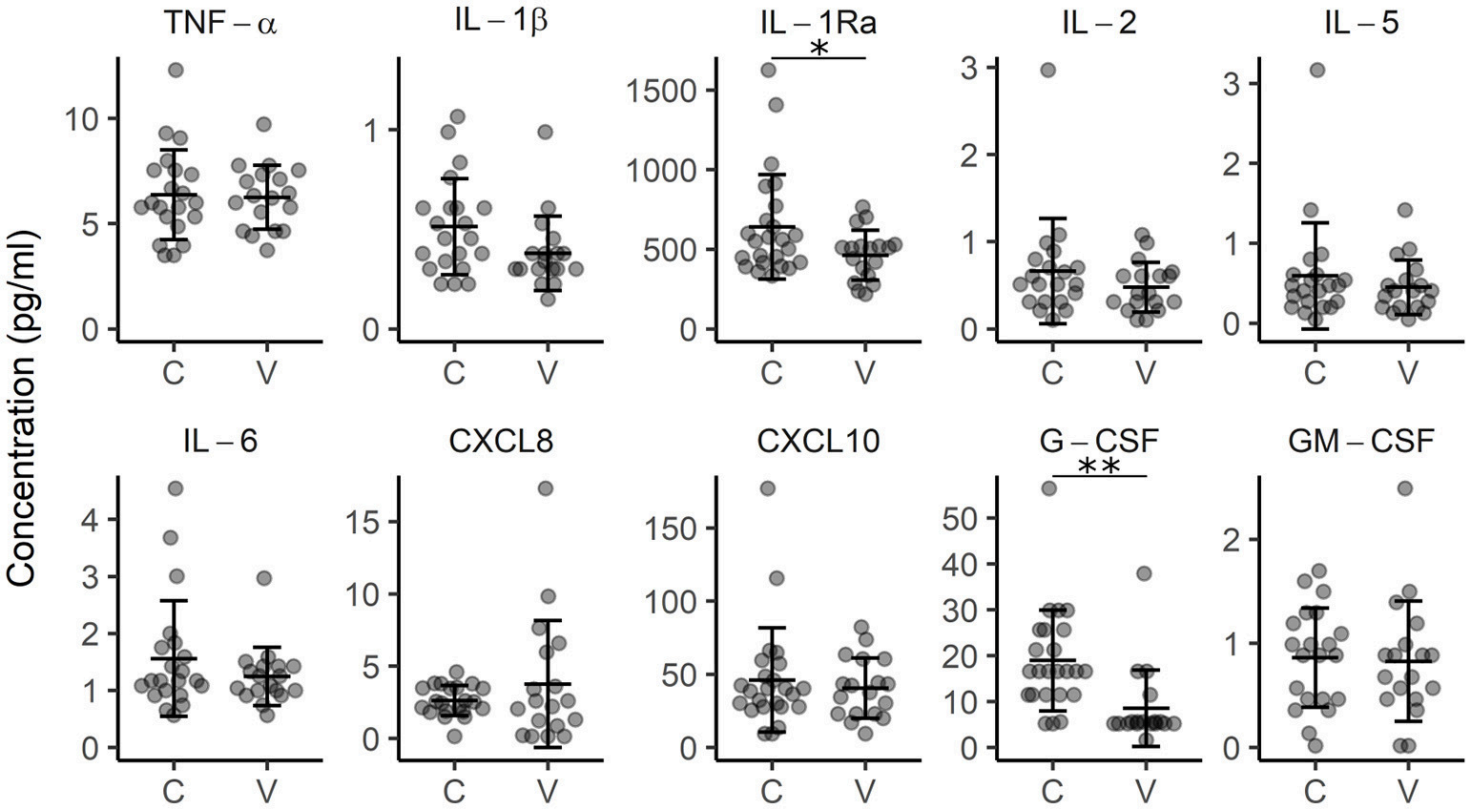
Concentration of cytokines in the plasma of control subjects (C) and vitiligo patients (V). Arithmetic mean ± standard deviation is indicated. *p < 0.05; **p < 0.01.

To check for the possible signs of systemic inflammation, we measured the concentration of inflammation-associated cytokines (TNF-α, IFN-γ, IL-1b, IL-1Ra, IL-2, IL-5, IL-6, CXCL8, CXCL10, G-CSF, and GM-CSF) in the plasma of vitiligo patients. We found slightly lower plasma concentrations of IL-1 inhibitory IL1-Ra (*p* < 0.05) in vitiligo patients compared to those in control subjects, and also less G-CSF (*p* < 0.01) (Figure 2). G-CSF may have pro-inflammatory or pro-repair influence depending on the context, and therefore the impact of this change is difficult to judge (26). To conclude, these results further support that strong systemic inflammation is probably not a part of vitiligo pathogenesis.

### Innate-like cells may be responsible for vitiligo lesions

**Figure 3:**
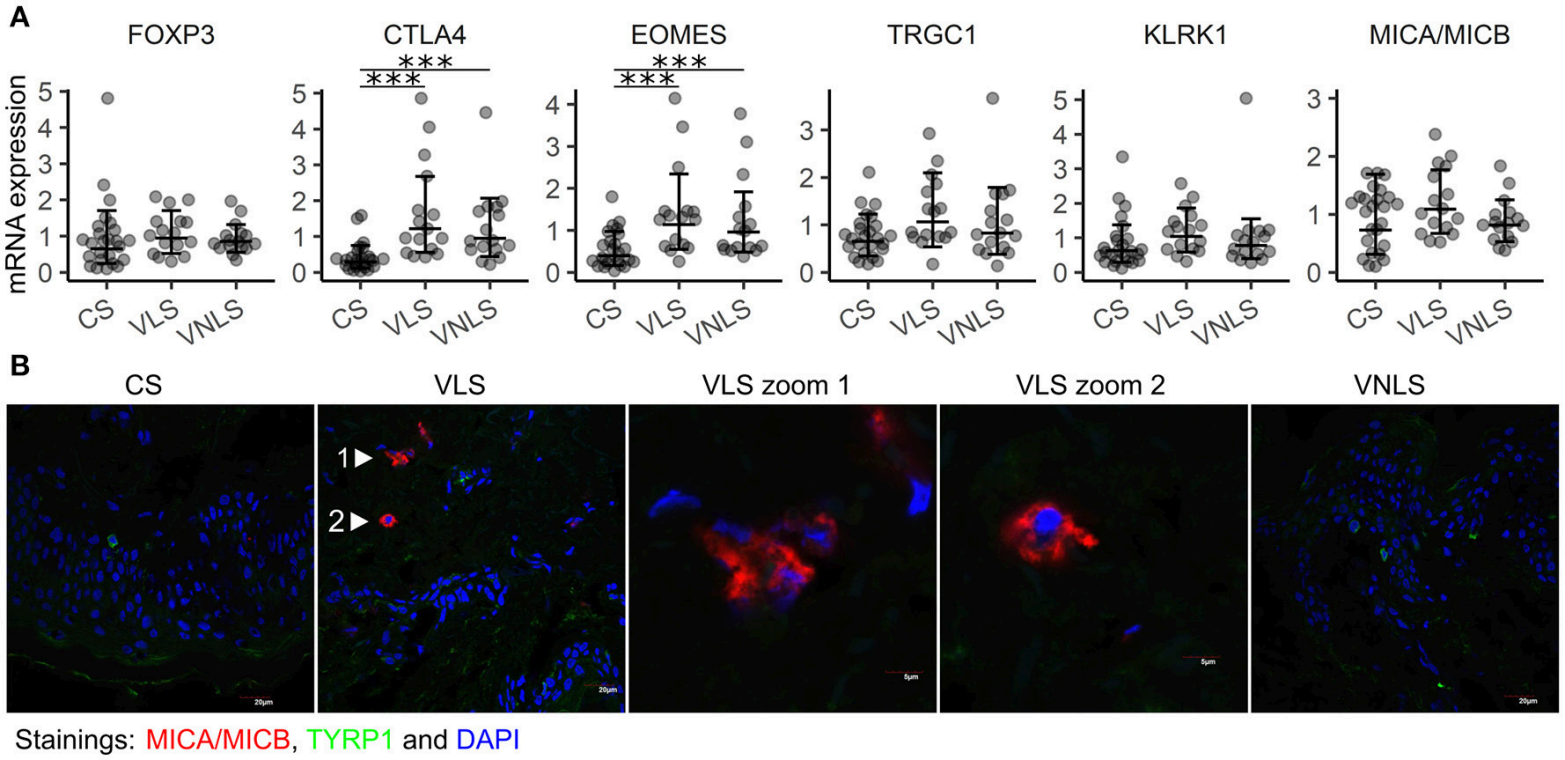
Innate-like cells and receptors are involved in vitiligo pathogenesis. **(A)** Relative mRNA expression of genes encoding markers of infiltrating immune cells and their target molecules. Geometric mean ×÷ geometric standard deviation is indicated. ***p < 0.001. **(B)** Immunofluorescence image of MICA/MICB (MHC class I chain-related protein A and B) and TYRP1 (tyrosinase related protein 1) in sections of biopsy samples from control subjects (CS), and in the lesional skin (VLS) and non-lesional skin (VNLS) of vitiligo patients. The images are representative of 3 healthy controls and 3 vitiligo patients.

Next, we analyzed immune cell infiltration to vitiligo lesions based on cell-type specific gene expression. In particular, we were interested in cells with cytotoxic potential toward melanocytes, and also in cells capable of counterbalancing immune responses. We did not find increased *FOXP3* levels in vitiligo skin, indicating comparable numbers of Tregs in the studied groups (Figure 3A). However, the expression of *CTLA4* was increased in vitiligo lesional and non-lesional skin (*p* < 0.001 and *p* < 0.001, respectively) (Figure 3A). As *CTLA4* is expressed in Tregs as well as in activated T cells, we suggest that *CTLA4* is upregulated in our samples due to increased activation of T cells in the skin of vitiligo patients. Moreover, *EOMES*, a transcription factor characteristic for effector cytotoxic T cells and innate and innate-like virtual memory T cells, was increased in both vitiligo lesional and non-lesional skin (*p* < 0.001 and *p* < 0.001, respectively) (Figure 3A) (27, 28). *KLRK1*, encoding the activating NK cell receptor NKG2D and *TRGC1* that encodes a T cell receptor γ-chain showed a tendency for increased expression in vitiligo lesional skin compared to that in healthy skin (*p* = 0.055 and *p* = 0.058, respectively) (Figure 3A). Notably, the stress molecule MHC class 1 chain-related protein A and B (MICA/MICB) that can be bound by activating NK cell receptors also showed a tendency for increased expression in vitiligo lesions (*p* = 0.052) (Figure 3A). Next we performed MICA/MICB immunofluorescence staining on sections from vitiligo and control biopsy samples together with the melanocyte marker TYRP1 (Figure 3B, Figure S1). While control skin and vitiligo non-lesional skin were completely clean of MICA/MICB, we noted multiple positive cells in the sub-epidermal area of vitiligo lesional skin. Often the stained cells were with disturbed nuclear morphology, as can be seen in the middle panel of Figure 3B. There was no co-localisation of MICA/MICB with TYRP1. The much more dramatic differences in MICA/MICB protein expression in comparison to gene expression data can be explained by the posttranscriptional and posttranslational regulation of stress molecules (29). To conclude, the data suggest the importance of stress-ligand upregulation in the dermis of vitiligo lesional skin and the presence of activated innate-like T cells at the center of the pathological process indicating the involvement of lymphoid stress surveillance responses in vitiligo pathogenesis.

### Active vitiligo is characterized by increased proportion of circulating memory B cells

**Figure 4:**
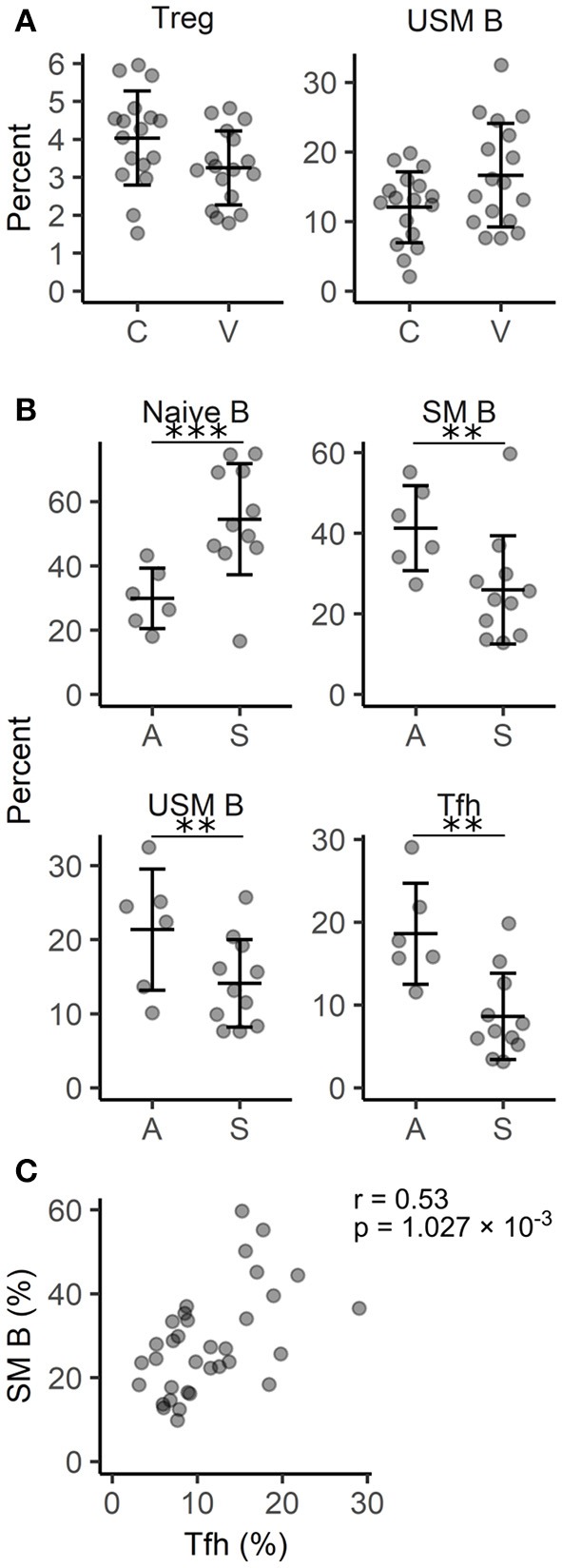
Comparison of circulating lymphocyte subpopulations in vitiligo patients with different disease activity. **(A)** The percentage of regulatory T cells (Treg) among T helpers and unswitched memory (USM) B cells among B cells in vitiligo patients (V) and control individuals (C). **(B)** The percentage of naïve, switched memory (SM) and USM B cells among B cells and follicular helper T cells (Tfh) among T helper cells in vitiligo patients with active (A) Vs. stable (S) disease. Arithmetic mean ± standard deviation is indicated. *p < 0.05; **p < 0.01; and ***p < 0.001. **(C)** A scatter plot showing the correlation between the percentages of SM B cells and Tfh cells. Pearson's correlation coefficient (r) and the *p*-value of the association test are indicated on the plot.

Next, we applied flow cytometry to find out whether any signs of immune cell dysregulation could be seen among the circulating lymphocyte subpopulations. The gating strategy for various subpopulations of classical and non-classical T cells as well as B cells is depicted in Figure S2. Using CCR7, CD45RA and CD28, CD4+ and CD8+ T cells were divided into subpopulations at different levels of maturation. These subpopulations as well as the percentages of Vδ1 TCR+ and Vδ2 TCR+ γδ T cells, mucosa associated invariant T cells, and NK cells did not demonstrate significant differences between the studied groups (Table S6). We could identify only a moderate decrease in the percentage of Tregs (*p* < 0.05) confirming previous findings (30) but also an increased proportion of unswitched memory (USM) B cells (Figure 4A). When vitiligo patients were divided into subgroups according to their disease activity, it was evident that active disease was associated with decreased percentages of naïve B cells and increased proportions of switched memory (SM) and USM B cells (Figure 4B, Table S7). The reduction of naïve B cells does not probably stem from impaired generation of B cells in the bone marrow as the percentages of transitional B cells mostly remained unchanged. Instead, activation of germinal centers and faster B cell isotype switching is supported by concomitant upregulation of T follicular helper (Tfh) cell percentages in patients with active vitiligo (Figures 4B,C). To conclude, our results point to the involvement of B cells and germinal center reactions in vitiligo pathogenesis, although the disease is believed to be mediated by cytotoxic T cells.

### *WIPI1* is significantly downregulated in vitiligo skin

**Figure 5:**
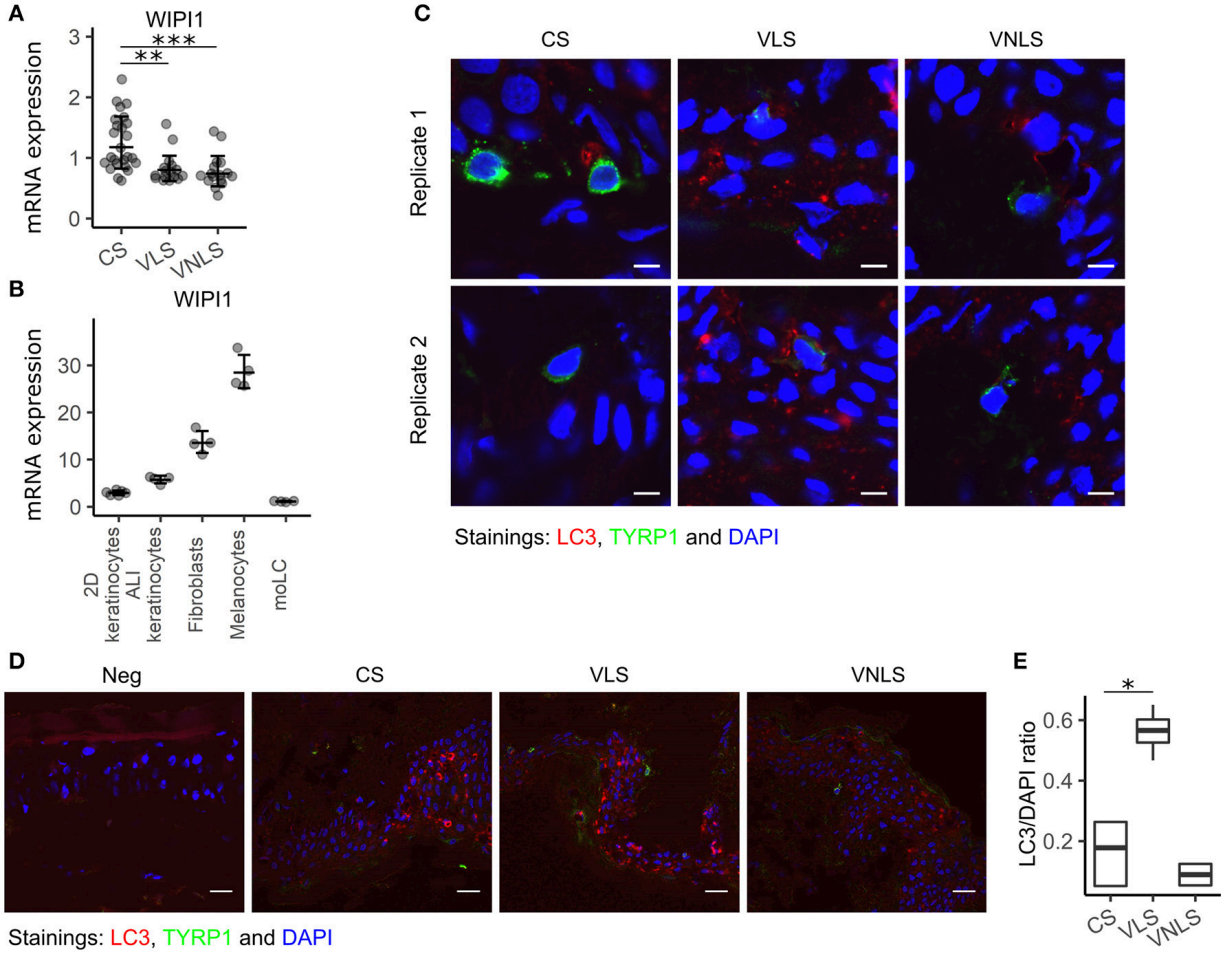
*WIPI1* expression and LC3 staining. **(A)** *WIPI1* relative mRNA expression in the skin of control subjects (CS), and in the perilesional skin (VLS) and non-lesional skin (VNLS) of vitiligo patients. Geometric mean ×÷ geometric standard deviation is indicated. **p < 0.01; and ***p < 0.001. **(B)** *WIPI1* relative mRNA expression in various cell types of the skin. Geometric mean ×÷ geometric standard deviation is indicated. 2D, two-dimensional; ALI, air-liquid interface; moLC, monocyte derived Langerhans cells. **(C)** Immunofluorescence image of LC3 (microtubule-associated protein 1A/1B-light chain 3) and TYRP1(tyrosinase related protein 1) in sections of biopsy samples from control subjects (CS), and lesional skin (VLS) and non-lesional skin (VNLS) of vitiligo patients. The white bar represents 5 μm. **(D)** Immunofluorescence image as in panel C with the white bar representing 20 μm. Neg. refers to a control slide with secondary antibodies only. **(E)** The box and whiskers plots show the median and interquartile range of the ratio between the fluorescence signal marking LC3 expression and DAPI (cell nuclei) in CS, VNLS, and VLS. Whiskers cover data points within a 1.5× interquartile range. *p < 0.05.

According to several hypotheses of vitiligo pathogenesis, melanocytes are not merely innocent bystanders that are attacked by cytotoxic cells, but rather trigger the processes themselves by impaired oxidative stress responses and/or impairment of autophagic processes. Therefore, we set out to study the expression of *WIPI1* that is a gene regulating autophagy and melanosome maturation (31, 32). Its expression was significantly downregulated in both lesional (*p* < 0.01) and non-lesional (*p* < 0.001) skin taken from patients with vitiligo (Figure 5A). We compared *WIPI1* mRNA expression levels in various cell types present in the skin and found the highest expression level in melanocytes that was followed by fibroblasts (Figure 5B). *WIPI1* expression was relatively low in monocyte derived Langerhans cells and keratinocytes in 2-dimensional culture but was increased during their differentiation at the air-liquid interface. Regarding the highest contribution of *WIPI1* expression from melanocytes in the biopsy samples, *WIPI1* downregulation could be the result of melanocyte loss in the lesions. However, this is unlikely regarding the reduced *WIPI1* expression in vitiligo non-lesional skin.

To determine if *WIPI1* downregulation in vitiligo skin can be associated with impaired autophagy in melanocytes, we stained the tissue sections of skin biopsy samples for microtubule-associated protein light chain 3 (LC3) (Figure 5C). When autophagy is initiated, the cytosolic form of LC3 is conjugated to phosphatidylethanolamine to form a lipidated LC3 conjugate, which is recruited to autophagosomal membranes. This is associated with the conversion of the dim homogenous fluorescence to a dotted pattern in the cytosol. In control skin and in vitiligo non-lesional skin, most of the melanocytes were devoid of LC3 dots, whereas in the vitiligo lesions, the scarce remaining melanocytes with weak TYRP1 staining showed an LC3 pattern characteristic for autophagy (Figure 5C, Figure S3). To our surprise, keratinocytes in vitiligo lesions also had significantly more autophagic vacuoles throughout all the layers of epidermis, in comparison to control and non-lesional skin (Figures 5D-E, Figure S4). Autophagy and nucleophagy is thought to be essential for normal epidermal development and differentiation (32). To the best of our knowledge, this is the first description of excessive autophagy in vitiligo keratinocytes. However, previous transcriptional studies have found alterations in keratinocytes from vitiligo lesions (7). This suggests that the cross-talk between different skin cell types is relevant in vitiligo pathogenesis. Collectively, our results are in line with increased autophagy in vitiligo lesions, involving melanocytes as well as keratinocytes.

## Discussion

This study provided many insights into vitiligo pathogenesis. The results suggest the participation of lymphoid immune surveillance in vitiligo lesions, activation of germinal center reactions in secondary lymphoid organs during lesion extension, and support the involvement of autophagy in disease development.

Vitiligo lesions develop without severe clinical signs of inflammation. In line with this, the comparative gene expression study of skin biopsy samples revealed only a mild inflammatory response in the marginal areas of vitiligo lesions where the destructive processes occur. Among the studied inflammatory chemokines, only CXCL10 stood out as a factor that is significantly upregulated right in the center of the pathological processes. The importance of CXCL10 together with closely related CXCL9 (33) has been substantiated by previous studies in humans as well as in mouse models of vitiligo (9, 23, 24, 34). Although CXCL10 is known as an IFN-γ-induced and Th1-associated chemokine, CXCL10 expression is also influenced by type I IFNs (35). Indeed, the tissue-resident memory T (Trm) cells in vitiligo skin express CXCR3, the receptor for CXCL10, and are prone to IFN-γ production (12, 14). However, the mRNA of type II IFN, IFN-γ, remained below the detection limit in our biopsy samples. In contrast, recent evidences suggest that type I IFNs play a role in vitiligo pathogenesis: IFN-α producing plasmacytoid dendritic cells infiltrate active vitiligo skin (16, 36); pegylated IFN-α2a and IFN-α2b, which are used in the treatment of chronic hepatitis C, induce depigmentation at the injection sites (37), and vitiligo patches have been observed at the site of application of imiquimod, a Toll-like receptor (TLR)-7 and TLR-8 agonist that enhances IFN-α production (16). In addition to CXCL10, IFN-induced *IFIH1* was upregulated in vitiligo skin in the current study. Moreover, it has been previously observed that the intracellular innate immune receptor IFIH1 induces type I IFNs and that IFIH1 is implicated in autoimmune processes (38), and is one of the susceptibility loci/risk genes for vitiligo (5). During the course of vitiligo, type I IFNs can be induced by the cGAS–cGAMP–STING pathway after oxidative stress generated DNA damage (39, 40). To conclude, the results of the current study indirectly support the association between type I IFNs and vitiligo. Nevertheless, the specific and/or overlapping roles of type I and II IFNs in vitiligo have yet to be substantiated.

Our study also helps establish the link between lymphoid stress surveillance response and vitiligo. In addition to microbes, tissue dysregulation can be sensed by lymphocytes via recognition of stress molecules (like MICA/MICB) that have been upregulated by damaging agents like oxidative stress (29, 41). In lymphoid stress surveillance, lymphocytes are rapidly activated through their nonclonotypic receptors and deploy their effector mechanisms like cytokine secretion and cytotoxic mediators without any delays (41, 42). We could clearly observe staining for MICA/MICB in dermal areas of vitiligo lesional skin but not in healthy or non-lesional skin. Although melanocytes are situated at the bottom layer of the epidermis, dermal changes in vitiligo have been described by previous studies as well (43). MICA/MICB are ligands for the activating NKG2D receptor, expressed by NK cells, γδ T cells, and a subpopulation of cytotoxic αβ T cells (42). Involvement of innate cells in vitiligo has been suggested by Yu et al. (44). We could not detect significant upregulation of *TRGC1* that encodes a T cell receptor γ-chain arguing against the specific recruitment of γδ T cells to vitiligo lesions. However, this is a tissue resident population of innate-like T cells that is already located and ready to respond to stress signals by neighboring cells. Accordingly, the lack of increase in their numbers does not mean that they are not responding to the stress-ligands. However, the increase in transcription factor *EOMES* is consistent with the involvement of innate-like virtual memory T cells that like innate cells, respond readily to cytokines and stress signals, and importantly, can be recruited by CXCL10 (27, 28). Interestingly, it has been reported that NKG2D-dependent communication between dysregulated epithelial cells and tissue-associated lymphoid cells also affects the systemic immune compartment in mice (42). In that study, epithelial stress-surveillance enhanced the response to antigens that were introduced together with epithelial stress, and provoked the T helper 2 and antibody responses linking lymphoid stress surveillance to atopy. These data from mouse models are echoed by clinical findings that atopic dermatitis is included among the vitiligo comorbidities (45). Moreover, in line with this information, we found significantly more switched and unswitched memory B cells and T follicular helpers in patients with active vitiligo compared to those in patients with stable disease (with good correlation between the percentages of SM B and Tfh). This suggests that although the damage is probably caused by local resident lymphocytes, the local processes may have more profound systemic effects than suspected so far. The involvement of humoral responses in vitiligo is corroborated by the finding of circulating autoantibodies directed toward melanocytic antigens whose levels correlate with disease activity as biomarkers of melanocyte destruction (46, 47). However, the precise cell type responsible for lymphoid stress surveillance in vitiligo and the molecular mechanisms that link the local processes in skin with systemic effects are yet to be identified.

Interestingly, we demonstrated the highly significant downregulation of a regulator of autophagy, *WIPI1* (32), in lesional and non-lesional skin of vitiligo patients. Autophagy is the enzymatic digestion of cytoplasmic contents. In this process, cytoplasmic proteins are trapped within vesicles called autophagosomes, which fuse with lysosomes and the cytoplasmic proteins are proteolytically degraded. Although autophagy is primarily a mechanism for degrading damaged cellular organelles and proteins to maintain cellular homeostasis, it is shown to be involved in pathological processes including autoimmunity, infections, and malignant tumors (48, 49). Autophagy is controlled by macromolecular signaling complexes. Of these, Beclin 1 with UV radiation resistance-associated tumor suppressor gene protein (UVRAG) are positive regulators, and the mammalian target of rapamycin (mTOR) is a negative regulator of autophagy (48). The idea that dysfunction of autophagy is involved in vitiligo pathogenesis is supported by Jeong et al., who found that variation in UVRAG gene contributes to the risk of non-segmental vitiligo in the Korean population (50), and by Wang et al., who demonstrated that several genes involved in the autophagy process were dysregulated in leukocytes of generalized vitiligo patients (51). Moreover, autophagy deficiency causes premature senescence and decreased proliferation of melanocytes (52), and melanosomal autophagy in stressed melanocytes mediates antigen presentation and dendritic cell maturation (53). However, melanosomes are also lysosome-related organelles whose maturation is controlled by molecules overlapping with autophagy regulators like WIPI1 and LC3 (31, 54). Therefore, in vitiligo skin, *WIPI1* downregulation may be rather linked with impaired maturation of melanosomes and not with impaired autophagy. According to our data, downregulation of *WIPI1* in vitiligo skin and activation of autophagy are uncoupled processes as *WIPI1* expression was reduced to a similar degree in lesional and non-lesional skin whereas LC3 staining clearly indicated increased autophagy only in keratinocytes of lesional skin. Whether the increased autophagy in vitiligo keratinocytes represents a compensatory mechanism for the lack of melanin, is responsible for the faster degradation of remaining melanin produced by residual melanocytes in vitiligo skin (55) or is the result of increased stress-surveillance response in the skin is still an open question.

Thus, we propose a model wherein different stressors like UV irradiation or dysregulated melanosome maturation lead to increased oxidative stress that can cause DNA damage. Damaged cells upregulate stress-ligands and type I IFN production. IFNs induce CXCL10 secretion from keratinocytes, which then attracts CXCR3 positive T cells. Stress-ligands can be bound by activating receptors on innate and innate-like T cells which unleash their effector mechanisms and cause autoantigen release from dying cells. This can prime antigen-specific T and B cell responses, which participate in the perpetuation of tissue damage.

## Ethics statement

This study was carried out in accordance with the recommendations of Research Ethics Committee of the University of Tartu. All subjects gave written informed consent in accordance with the Declaration of Helsinki. The protocol was approved by the Research Ethics Committee of the University of Tartu.

## Author contributions

LR, EP, and MP performed and analyzed the gene expression experiments, LR and MS performed the bioinformatic analysis of the data and prepared the figures, EK and MŠ designed and performed the flow cytometric experiments, HV performed immunofluorescence staining, LR and MP carried out Luminex experiments. LR, KüK, MK and KA sampled the patients and collected the clinical data. KaK, KüK, PP and AR supervised research and reviewed data. LR and KaK wrote the paper with contributions from all authors.

### Conflict of interest statement

The authors declare that the research was conducted in the absence of any commercial or financial relationships that could be construed as a potential conflict of interest.
