# Supplementary material for: Lymphoid Stress Surveillance Response Contributes to Vitiligo Pathogenesis
Source: Front Immunol. 2018 Nov 20;9:2707. doi: 10.3389/fimmu.2018.02707 (PMC6255962; doi:10.3389/fimmu.2018.02707)

Supplementary material

Lymphoid stress surveillance response contributes to vitiligo pathogenesis

Liisi Raam[1, 2], Epp Kaleviste[3], Marina Šunina[3], Helen Vaher[3], Mario Saare[3], Ele Prans[3], Maire Pihlap[3], Kristi Abram[1, 2], Maire Karelson[1, 2], Pärt Peterson[3], Ana Rebane[3], Kai Kisand[3,4*], Külli Kingo[1, 2,4*]

[1] Department of Dermatology, University of Tartu, Tartu, Estonia

[2] Dermatology Clinic, Tartu University Hospital, Tartu, Estonia

[3] Institute of Biomedicine and Translational Medicine, University of Tartu, Tartu, Estonia

[4] Shared corresponding author

*** Correspondence:**

Kai Kisand

Kai.kisand@ut.ee

Külli Kingo

Kylli.Kingo@kliinikum.ee

Table S1. Characteristics of study participants.

	Control subjects	Vitiligo patients
Number of participants	28	21
Male/female	8/20	6/15
Age range (y)	24-57	19-60
Mean age (y)	36.3	37.4
Mean age of the onset of vitiligo (y)		21.8
Active/stable vitiligo	–	7/14
Body surface area affected by vitiligo <10%/≥10%		9/12

Table S2. Primer sequences		
Gene	Forward primer	Reverse primer
ACTB – actin beta	CTGGAACGGTGAAGGTGACA	CGGCCACATTGTGAACTTTG
AIM2 – absent in melanoma 2	TAAGTCAAGCTGAAATGAGTCCTG	TTCTGTTCAGGCTTAACATGAGG
CASP1 – caspase 1	AAAGTCGGCAGAGATTTATCCA	GATGTCAACCTCAGCTCCAG
CCL2 – C-C motif chemokine ligand 2	AAGCTGTGATCTTCAAGACC	GTTCAAGTCTTCGGAGTTTGG
CCL5 - C-C motif chemokine ligand 5	CCATATTCCTCGGACACCAC	TTTCGGGTGACAAAGACGAC
CCL20 - C-C motif chemokine ligand 20	ATCAGAAGCAAGCAACTTTGAC	TGATAGCATTGATGTCACAGCC
CCL27 - C-C motif chemokine ligand 27	AGAGAAGGAAGAGTCTAGGCTG	GTAGGAATGCTGCTGTAGGG
CTLA4 – cytotoxic T-lymphocyte associated protein 4	TGAACCTGGCTACCAGGACC	CCACGTGCATTGCTTTGC
CXCL1 – C-X-C motif chemokine ligand 1	ATGCTGAACAGTGACAAATCC	CTCAAACACATTAGGCACAATCC
CXCL2 – C-X-C motif chemokine ligand 2	CCAAACCGAAGTCATAGCCACAC	GCCACCAATAAGCTTCCTCCT
CXCL8 – C-X-C motif chemokine ligand 8	GAGAGTGATTGAGAGTGGAC	GAATTCTCAGCCCTCTTCAAA
CXCL10 – C-X-C motif chemokine ligand 10	TTCCTGCAAGCCAATTTTGT	TTCTTGATGGCCTTCGATTC
EOMES - eomesodermin	GACAACTATGATTCATCCCATCAG	GGCTTGAGGTAAAGTGTTGAC
FOXP3 – forkhead box 3	TGGAGAAGGAGAAGCTGAGTGC	CCCTTGTCGGATGATGCCA
IFIH1 – interferon induced with helicase C domain 1	ATGGAGCAATATACTAGGACTG	TTGTTCATTCTGTGTCATGG
IFNAR1 - Interferon alpha and beta receptor subunit 1	CCATCCAAAGCCCACATAACACT	CGCAAAGCTCAGATTGGTCCT
IFNG – interferon gamma	TTCGGTAACTGACTTGAATGTCCA	TTTCGCTTCCCTGTTTTAGCTG
IFNGR – interferon gamma receptor 1	GCTCGTCTCCATTTACAAAAACTGA	GGAAAAATTGGACCACCTAAACTG
IL17A – interleukin 17A	ACCGCAATGAGGACCCTGAGA	TCCACGTTCCCATCAGCGT
IL17F – interleukin 17F	GGAATTACACTGTCACTTGG	GATGTCTTCCTTTCCTTGAG
IL1B – interleukin 1 beta	AAACAGATGAAGTGCTCCTTCC	CATCTTCCTCAGCTTGTCCA
IL36A – interleukin 36, alpha	GCCGTCTATCAATCAATGTGTA	ACTGTCACTTCGTGGAACTG
IL1RN – interleukin receptor antagonist	AAGATAGATGTGGTACCCATTGAG	TGATGTTAACTGCCTCCAGC
IL20RA – interleukin 20 receptor subunit alpha	GAACCTACTGTGATCTTTCTGCT	GTGGGCCAATTTGTGTTTCTAA

IL22 – interleukin 22	AGCACATGTCATATTGAAGG	GTTCTCCAATTGCTTTGATCTC
IL22RA1 – interleukin 22 receptor subunit alpha 1	ATCGAGTATAAGACGTACGGAG	CATAGTAGAGCTCCGTGAGG
IL22RA2 - interleukin 22 receptor subunit alpha 2	TGGTGTAGCAGGAACTCAGTC	TGCTGTTGCCAGTAAGTGCC
KLRK1 – killer cell lektin like receptor K1	TATACAGCAAAGAGGACCAGGA	TATTGTTAGTAGGTTGGGTGAGAG
MICB – MHC class I polypeptide-related sequence B	CTATGACAGGCAGAAACGCA	GAATGCAAGCCTCCTTTCTG
NLRP1 – NLR family pyrin domain containing 1	ACTTGTACCGAGTTCACTTCC	CTGTGCTGTGGGTTGATCTC
NLRP3 - – NLR family pyrin domain containing 3	ACATGCCCAAGGAGGAAGAG	GGCTGTTCACCAATCCATGAG
PYCARD – PYD and CARD domain containing	CTGGTCAGCTTCTACCTGGA	CTATAAAGTGCAGGCCTGGC
TNF – tumor necrosis factor	CCCATGTTGTAGCAAACCCT	AGGACCTGGGAGTAGATGAG
TRGC1 – T cell receptor gamma constant 1	GGACAAAGAACACAGATGTATCGT	GCAGCAGTAGTGTATCATTTGCA
WIPI1 – WD repeat domain, phosohoinositide interacting 1	TTTCCTCCTATAATCTTGTGCCGT	ACTCCATTCTTCCGCCTTCC

Table S3. Used antibodies.

Panel 1: T cell subpopulations

Antibody and dye	Manufacturer	Clone
CD25 FITC	BioLegend	BC96
HLA-DR PerCP-Cy5.5	BioLegend	L234
CD31 APC	BioLegend	WM59
CD4 Alexa Fluor 700	BioLegend	OKT4
CD127 biotin	eBioscience	eBioRDR5
Brilliant Violet 421 Streptavidin	BioLegend	-
CD27 Brilliant Violet 510	BioLegend	O323
PD1 Brilliant Violet 605	BioLegend	EH12.2H7
CD3 Brilliant Violet 650	BioLegend	OKT3
CD57 PE	BioLegend	HNK-1
CD197 (CCR7) PE-Dazzle 594	BioLegend	G043H7
CD28 PE-Cy5	BioLegend	CD28.2
CD45RA PE-Cy7	BioLegend	HI100
CD8 BUV395	BD Biosciences	RPA-T8

Panel 2: NK cells and unconventional T cells

Antibody and dye	Manufacturer	Clone
TCR Vd1 FITC	Thermo	TS8.2
CD161 PerCP-Cy5.5	BioLegend	HP-3G10
CD4 APC	BioLegend	RPA-T4
CD16 Alexa Fluor 700	BioLegend	3G8
CD56 APC-Cy7	BioLegend	HCD56
CXCR5 Brilliant Violet 421	BioLegend	J252D4
TCR va7 Brilliant Violet 510	BioLegend	3C10
CD8 Brilliant Violet 605	BioLegend	RPA-T8
CD3 Brilliant Violet 650	BioLegend	OKT3
TCR Vd2 PE	BioLegend	B6
CD197 (CCR7) PE-CF594	BioLegend	150503
TCR Va24-Ja18 PE-Cy7	BioLegend	6B11

Panel 3: B cells

Antibody and dye	Manufacturer	Clone
CD95 Alexa Fluor 488	BioLegend	DX2
HLA-DR PerCP-Cy5.5	BioLegend	L234
CD19 APC	BioLegend	HIB19
IgD APC-Cy7	BioLegend	IA6-2
CD10 Brilliant Violet 421	BioLegend	HI10a
IgM Brilliant Violet 510	BioLegend	MHM-88
CD27 Brilliant Violet 605	BioLegend	O323
B220 Brilliant Violet 650	BioLegend	RA3-6B2
CD38 Brilliant Violet 711	BioLegend	HIT2
CD24 PE-CF594	BioLegend	ML5
CD3 PE-Cy5	BioLegend	UCHT1
CD14 PE-Cy5	BioLegend	61D3
CD56 PE-Cy5	BioLegend	HCD56
CD1d PE-Cy7	BioLegend	51.1

Table S4. Optical detectors configuration of flow cytometer.

Laser	PMT	LP Mirror	BP Filter	Intended Dye
488-nm blue laser	A	685	710/50	PerCP-Cy5.5
	B	505	530/30	FITC
	C		488/10	Side scatter (SSC)
405-nm violet laser	A	670	710/40	Brilliant Violet 711™
	B	630	670/30	-
	C	600	610/20	Brilliant Violet 605™
	D	535	540/30	-
	E	505	525/50	Brilliant Violet 510™
	F	-	440/40	-
561-nm YG laser	A	750	780/60	PE-Cy7
	B	685	710/50	-
	C	635	670/30	PE-Cy5
	D	600	610/20	PE-CF594
	E	-	586/15	PE
355-nm UV laser	A	505	530/30	-
	B	-	450/50	LIVE/DEAD®
640-nm red laser	A	750	780/60	APC-Cy7
	B	690	730/45	Alexa Fluor® 700
	C	-	670/14	APC

Table S5. Comparison of gene expression in the skin of patients with active or stable vitiligo. The mean and standard deviation (SD) are in log2 scale.

Gene name	Skin sample	Active vitiligo (mean (SD))	Stable vitiligo (mean (SD))	Active vs Stable (p-value)
TNFA	VLS	-0.058 (0.305)	-0.134 (0.736)	0.815
TNFA	VNLS	-0.369 (0.63)	0.048 (0.517)	0.172
IL1B	VLS	-1.177 (1.995)	-1.053 (1.525)	0.89
IL1B	VNLS	-1.208 (0.812)	0.126 (0.951)	0.013
IL1F6	VLS	-0.153 (0.666)	-0.113 (0.612)	0.905
IL1F6	VNLS	-0.018 (0.579)	-0.206 (0.82)	0.631
IL1RN	VLS	0.241 (0.395)	-0.251 (0.378)	0.026
IL1RN	VNLS	-0.027 (0.311)	-0.1 (0.551)	0.774
CCL2	VLS	-0.354 (0.441)	-0.037 (0.7)	0.339
CCL2	VNLS	-0.668 (0.696)	0.172 (0.455)	0.011
CCL5	VLS	-0.296 (0.816)	-0.219 (0.829)	0.859
CCL5	VNLS	-1.694 (0.642)	-0.138 (1.341)	0.019
CCL20	VLS	-0.286 (1.568)	-0.925 (1.394)	0.411
CCL20	VNLS	-0.689 (2.048)	-0.768 (1.644)	0.933
CCL27	VLS	0.179 (1.004)	-0.401 (0.553)	0.154
CCL27	VNLS	0.132 (0.459)	-0.201 (0.495)	0.203
CXCL1	VLS	-0.826 (0.978)	-0.022 (0.891)	0.114

CXCL1	VNLS	-0.985 (1.259)	-0.282 (1.32)	0.313
CXCL2	VLS	-0.84 (1.911)	-0.236 (1.015)	0.418
CXCL2	VNLS	-0.286 (0.942)	-0.592 (1.817)	0.71
CXCL8	VLS	-0.519 (1.667)	-1.186 (1.905)	0.49
CXCL8	VNLS	-2.657 (3.123)	-0.33 (1.729)	0.082
CXCL10	VLS	-0.64 (1.921)	-1.445 (2.222)	0.474
CXCL10	VNLS	-2.929 (1.713)	-1.351 (2.414)	0.184
IFIH1	VLS	-0.275 (0.859)	-0.085 (0.635)	0.619
IFIH1	VNLS	-0.314 (0.893)	-0.098 (0.696)	0.595
IFNAR1	VLS	0.02 (0.199)	-0.064 (0.38)	0.627
IFNAR1	VNLS	0.026 (0.332)	-0.092 (0.423)	0.569
IFNGR	VLS	-0.022 (0.379)	-0.066 (0.422)	0.838
IFNGR	VNLS	0.051 (0.326)	-0.094 (0.369)	0.442
IL22RA1	VLS	-0.013 (0.927)	-0.262 (0.73)	0.559
IL22RA1	VNLS	0.219 (0.373)	-0.421 (1.035)	0.171
IL22RA2	VLS	-0.068 (1.349)	-0.523 (1.073)	0.467
IL22RA2	VNLS	-0.316 (0.568)	-0.229 (1.138)	0.866
IL20RA	VLS	-0.011 (0.565)	-0.157 (0.587)	0.633
IL20RA	VNLS	-0.369 (0.417)	0.019 (0.721)	0.253
NLRP1	VLS	-0.163 (0.834)	-0.044 (0.369)	0.699
NLRP1	VNLS	-0.102 (0.385)	-0.016 (0.403)	0.68
NLRP3	VLS	-0.144 (1.405)	-0.966 (1.638)	0.324
NLRP3	VNLS	-0.684 (1.169)	-0.745 (1.851)	0.944
AIM2	VLS	-0.082 (1.07)	-0.421 (0.853)	0.507
AIM2	VNLS	-0.49 (1.436)	-0.238 (0.617)	0.629
PYCARD	VLS	0.15 (0.833)	-0.297 (0.215)	0.123
PYCARD	VNLS	-0.043 (0.685)	-0.118 (0.446)	0.793
CASP1	VLS	-0.058 (0.34)	0.003 (0.188)	0.649
CASP1	VNLS	-0.365 (0.529)	0.095 (0.44)	0.081

Table S6. Comparison of the mean cell percentages in the blood of healthy controls and vitiligo patients. SD, standard deviation.

Cell type/parent population	Control group (mean (SD))	Vitiligo group (mean (SD))	Control vs Vitiligo (p-value)
Th/T	64.186 (8.69)	62.711 (10.468)	0.658
Treg/Th	4.036 (1.244)	3.249 (0.976)	0.049
NV_Treg/Treg	24.191 (8.987)	23.814 (12.933)	0.922
RTE_Treg/Treg	10.596 (4.458)	11.418 (6.708)	0.677
CM_Treg/Treg	24.416 (5.155)	27.505 (6.391)	0.131
EM_Treg/Treg	45.694 (11.035)	46.018 (13.498)	0.939
NV_Th/Th	46.855 (15.729)	51.928 (12.079)	0.299
RTE_Th/Th	27.874 (13.088)	32.471 (11.882)	0.292
CM_Th/Th	25.222 (9.256)	26.665 (8.938)	0.647
EM_Th/Th	23.386 (10.74)	18.385 (8.406)	0.14

Themra/Th	3.559 (4.171)	2.199 (2.291)	0.247
CTL/T	29.235 (7.373)	30.725 (9.633)	0.616
NV_CTL/CTL	36.433 (15.744)	43.071 (15.534)	0.225
RTE_CTL/CTL	34.877 (15.501)	40.066 (16.002)	0.344
CM_CTL/CTL	7.429 (5.608)	6.543 (5.042)	0.631
EM_CTL/CTL	27.559 (18.153)	23.163 (13.414)	0.428
CTLemra/CTL	25.738 (16.98)	25.042 (17.038)	0.906
CD28neg_CTLemra/CTL	19.164 (13.258)	19.306 (15.001)	0.977

Th- T helper (CD4+ CD3+), Treg – regulatory T cell (CD4+ Cd25+ CD127lo), NV- naïve (CCR7+, CD45RA+), RTE – recent thymic emigrant (CD31+ NV), CM – central memory (CCR7+ CD45RA-), EM – effector memory (CCR- CD45RA-), emra – effector memory CD45RA+ (CCR7- CD45RA+)

B/ly	8.654 (3.161)	8.84 (3.981)	0.881
NV_B/B	56.68 (15.23)	45.839 (19.02)	0.076
SM_B/B	25.169 (9.789)	31.365 (14.302)	0.15
USM_B/B	12.059 (5.094)	16.666 (7.459)	0.043
Transitional/B	6.297 (3.248)	5.836 (4.337)	0.728
CD10pos_Transitional/B	3.949 (2.517)	3.826 (3.301)	0.904

Naïve B (IgD+ CD27-), SM – switched memory (IgD- CD27+), USM – unswiched memory (IgD+ CD27+), Transitional (CD24hi CD38 hi naïve)

Vd2/T	3.365 (3.078)	3.028 (2.143)	0.714
Vd1/T	0.829 (0.887)	1.294 (1.998)	0.387
Va24/T	0.137 (0.12)	0.118 (0.087)	0.593
MAIT/T	1.402 (1.436)	1.885 (1.382)	0.325
Tfh/Th	10.596 (4.238)	12.142 (7.263)	0.454
NK/ly	11.825 (4.16)	13.588 (7.105)	0.384

Vd2 – Vδ2 TCR+ γδ T cell (Vδ2 TCR+ CD3+), Vd1 – Vδ1 TCR+ γδ T cell (Vδ1 TCR+ CD3+), Va24 – NK T cell (Vα24+ CD3+), MAIT – mucosa associated invariant T cell (Vα7+ CD161+ CD3+), Tfh – T follicular helper (CD4+ CXCR5+), NK cell (CD3- CD16+ CD56+)

Table S7. Comparison of the mean cell percentages in the blood of patients with active or stable vitiligo. SD, standard deviation.

Cell type/parent population	Active vitiligo (mean (SD))	Stable vitiligo (mean (SD))	Active vs Stable (p-value)
CD4pos/T	60.992 (5.698)	63.648 (12.505)	0.494
Treg/Th	3.067 (0.976)	3.349 (1.008)	0.081
NV_Treg/Treg	26.128 (15.083)	22.552 (12.202)	0.719
RTE_Treg/Treg	12.462 (7.142)	10.848 (6.744)	0.5
CM_Treg/Treg	26.195 (6.165)	28.219 (6.69)	0.527
EM_Treg/Treg	45.153 (16.364)	46.49 (12.526)	0.928
NV_Th/Th	51.707 (19.081)	52.049 (7.166)	0.479
RTE_Th/Th	32.295 (16.672)	32.567 (9.32)	0.469
CM_Th/Th	26.503 (12.455)	26.754 (7.087)	0.772

EM_Th/Th	19.595 (12.36)	17.725 (5.944)	0.42
Themra/Th	1.365 (0.921)	2.654 (2.707)	0.182
CD8pos/T	32.655 (5.141)	29.673 (11.481)	0.411
NV_CTL/CTL	46.385 (21.1)	41.264 (12.381)	0.194
RTE_CTL/CTL	44.01 (21.557)	37.915 (12.764)	0.234
CM_CTL/CTL	2.915 (1.174)	8.522 (5.271)	0.069
EM_CTL/CTL	19.382 (15.229)	25.225 (12.597)	0.293
CTLemra/CTL	29.193 (12.858)	22.778 (19.126)	0.674
CD28neg_CTLemra/CTL	22.655 (14.422)	17.48 (15.673)	0.61

Th- T helper (CD4+ CD3+), Treg – regulatory T cell (CD4+ Cd25+ CD127lo), NV- naïve (CCR7+, CD45RA+), RTE – recent thymic emigrant (CD31+ NV), CM – central memory (CCR7+ CD45RA-), EM – effector memory (CCR- CD45RA-), emra – effector memory CD45RA+ (CCR7- CD45RA+)

B/ly	8.385 (3.504)	9.088 (4.363)	0.877
naive_B/B	29.893 (9.387)	54.537 (17.29)	0.001
SM_B/B	41.273 (10.525)	25.96 (13.449)	0.005
USM_B/B	21.372 (8.195)	14.1 (5.909)	0.002
Transitional/B	2.932 (1.482)	7.421 (4.602)	0.055
CD10pos_Transitional/B	2.118 (1.233)	4.758 (3.737)	0.183

Naïve B (IgD+ CD27-), SM – switched memory (IgD- CD27+), USM – unswiched memory (IgD+ CD27+), Transitional (CD24hi CD38 hi naïve)

Vd2/T	3.03 (2.061)	3.027 (2.285)	0.795
Vd1/T	1.467 (1.622)	1.2 (2.247)	0.399
Va24/T	0.112 (0.041)	0.121 (0.106)	0.619
MAIT/T	1.912 (0.711)	1.87 (1.674)	0.459
Tfh/Th	18.598 (6.107)	8.621 (5.207)	0.002
NK/ly	15.113 (8.681)	12.755 (6.397)	0.246

Vd2 – Vδ2 TCR+ γδ T cell (Vδ2 TCR+ CD3+), Vd1 – Vδ1 TCR+ γδ T cell (Vδ1 TCR+ CD3+), Va24 – NK T cell (Vα24+ CD3+), MAIT – mucosa associated invariant T cell (Vα7+ CD161+ CD3+), Tfh – T follicular helper (CD4+ CXCR5+), NK cell (CD3- CD16+ CD56+)

Figure S1. Split channels view of the images on Figure 3B

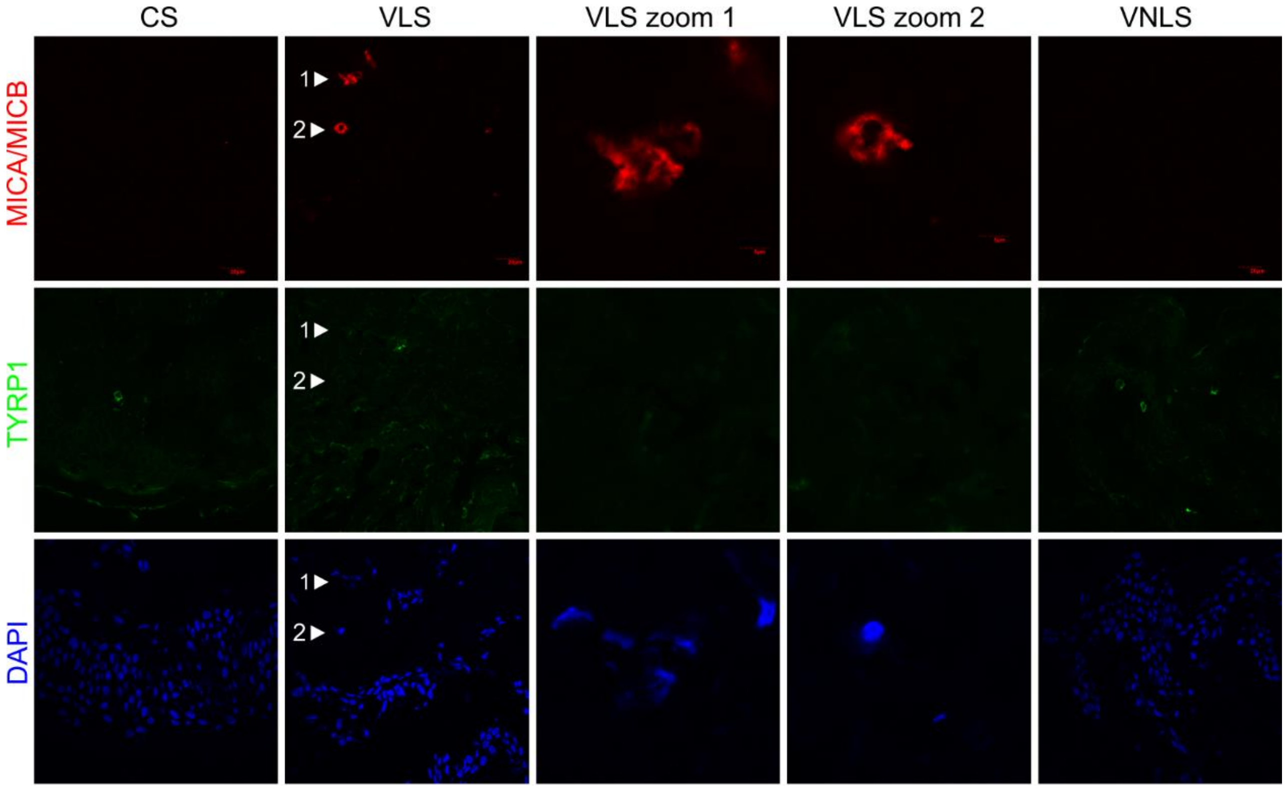
CS
VLS
VLS zoom 1
VLS zoom 2
VNLS
MICA/MICB
TYRP1
DAPI
1
2

Figure S2. Gating strategy for lymphocyte subpopulations

Panel 1:

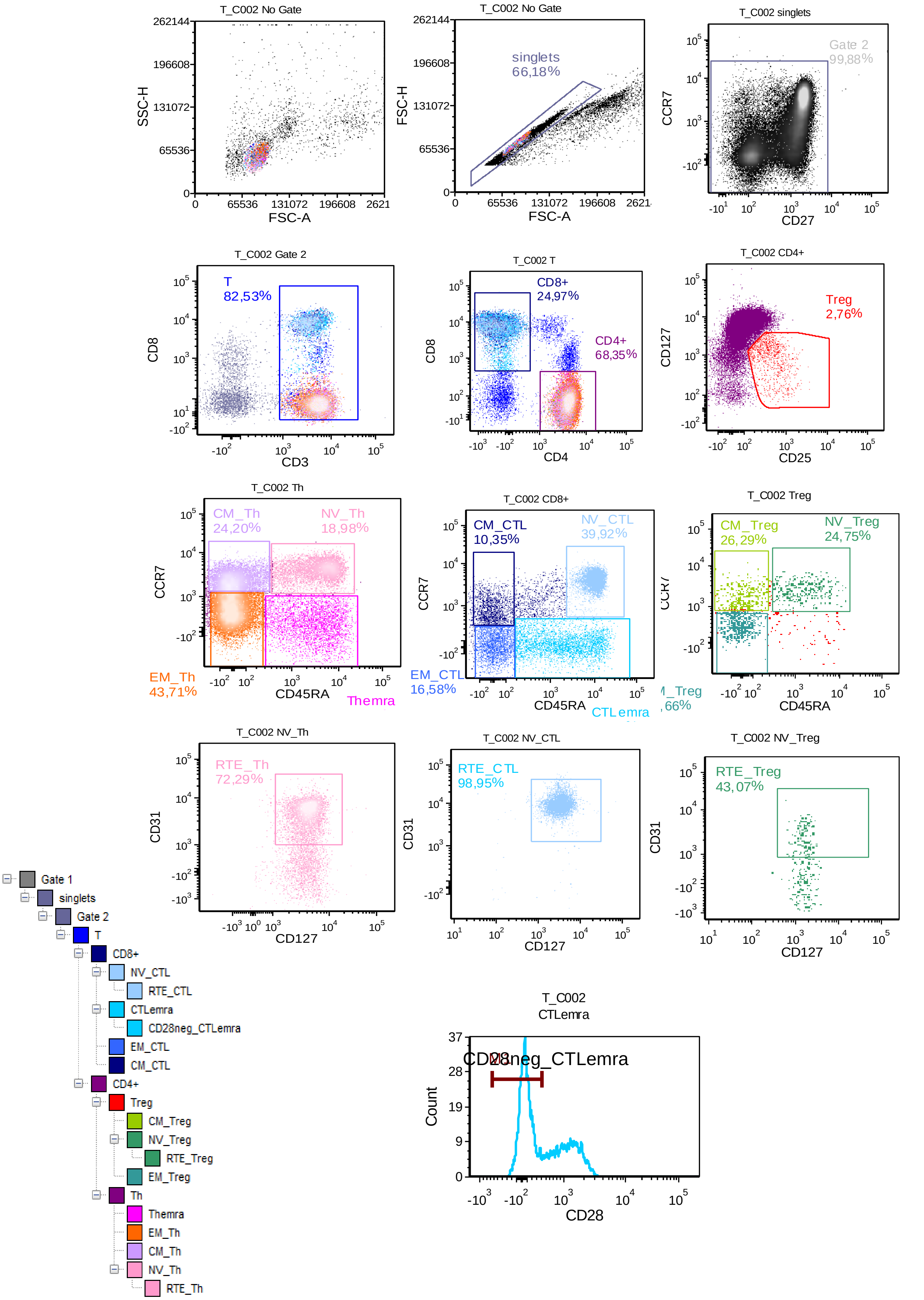

Panel 2:

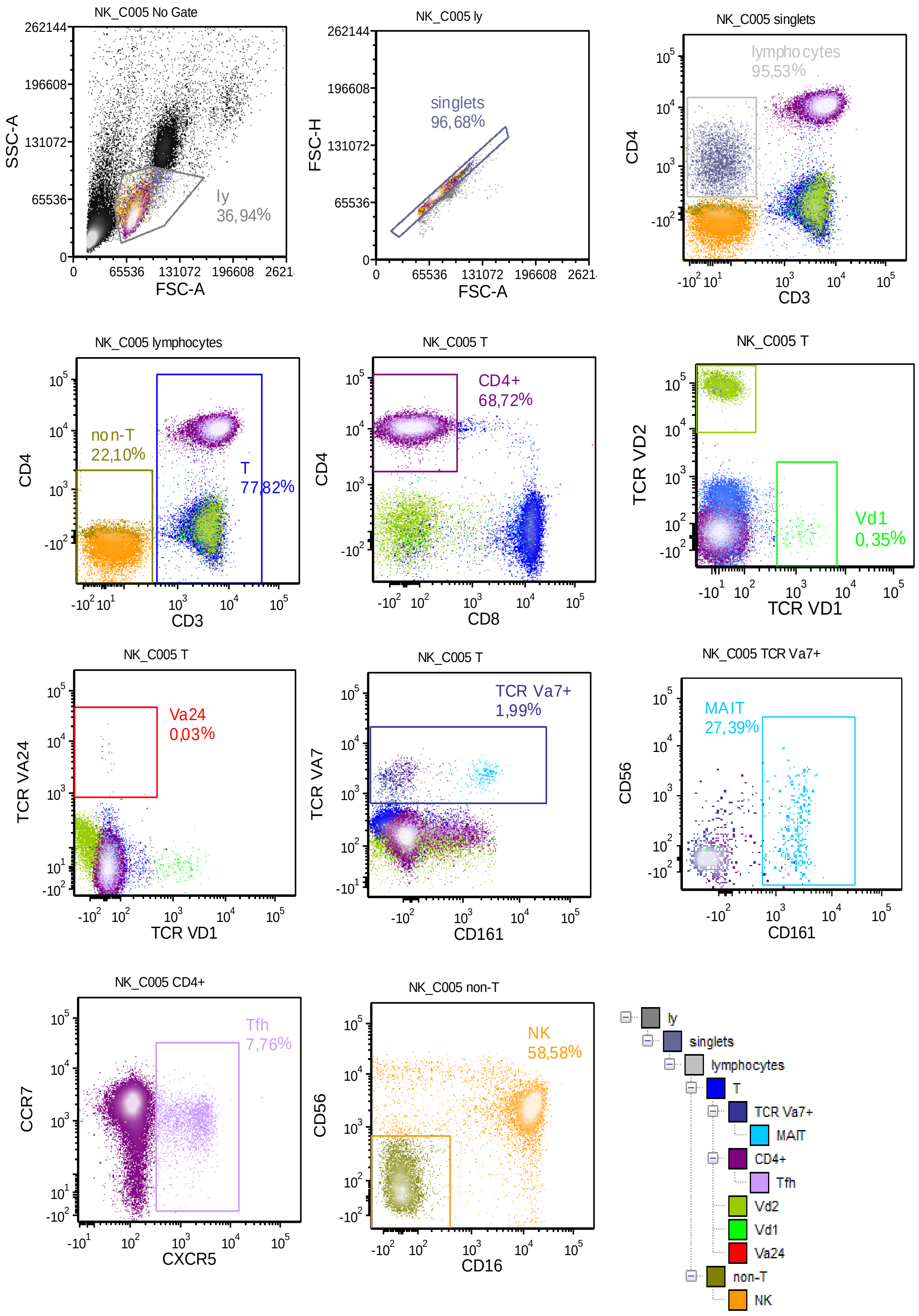

NK_C005 No Gate
SSC-A
FSC-A
ly
36,94%
NK_C005 ly
FSC-H
FSC-A
singlets
96,68%
NK_C005 singlets
CD4
CD3
lymphocytes
95,53%
NK_C005 lymphocytes
CD4
CD3
non-T
22,10%
T
77,82%
NK_C005 T
CD4
CD8
CD4+
68,72%
NK_C005 T
TCR VD2
TCR VD1
Vd1
0,35%
NK_C005 T
TCR VA24
TCR VD1
Va24
0,03%
NK_C005 T
TCR VA7
CD161
TCR Va7+
1,99%
NK_C005 TCR Va7+
CD56
CD161
MAIT
27,39%
NK_C005 CD4+
CCR7
CXCR5
Tfh
7,76%
NK_C005 non-T
CD56
CD16
NK
58,58%
ly
singlets
lymphocytes
T
TCR Va7+
MAIT
CD4+
Tfh
Vd2
Vd1
Va24
non-T
NK

Panel 3:

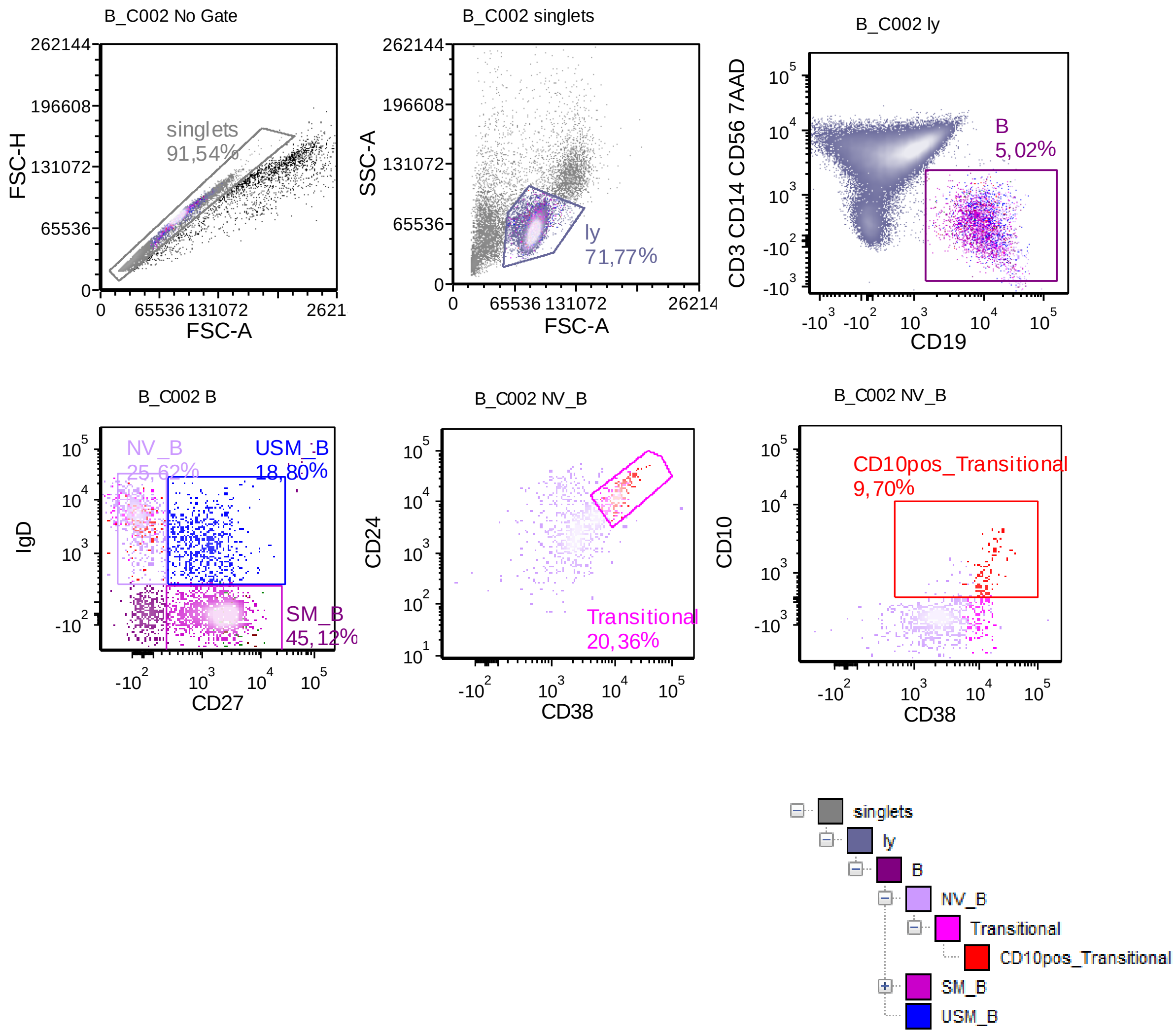

B_C002 No Gate
singlets
91,54%
FSC-H
FSC-A
B_C002 singlets
ly
71,77%
SSC-A
FSC-A
B_C002 ly
B
5,02%
CD3 CD14 CD56 7AAD
CD19
B_C002 B
NV_B
25,62%
USM_B
18,80%
SM_B
45,12%
IgD
CD27
B_C002 NV_B
Transitional
20,36%
CD24
CD38
B_C002 NV_B
CD10pos_Transitional
9,70%
CD10
CD38
singlets
ly
B
NV_B
Transitional
CD10pos_Transitional
SM_B
USM_B

Figure S3. Split channels view of the images on Figure 5C

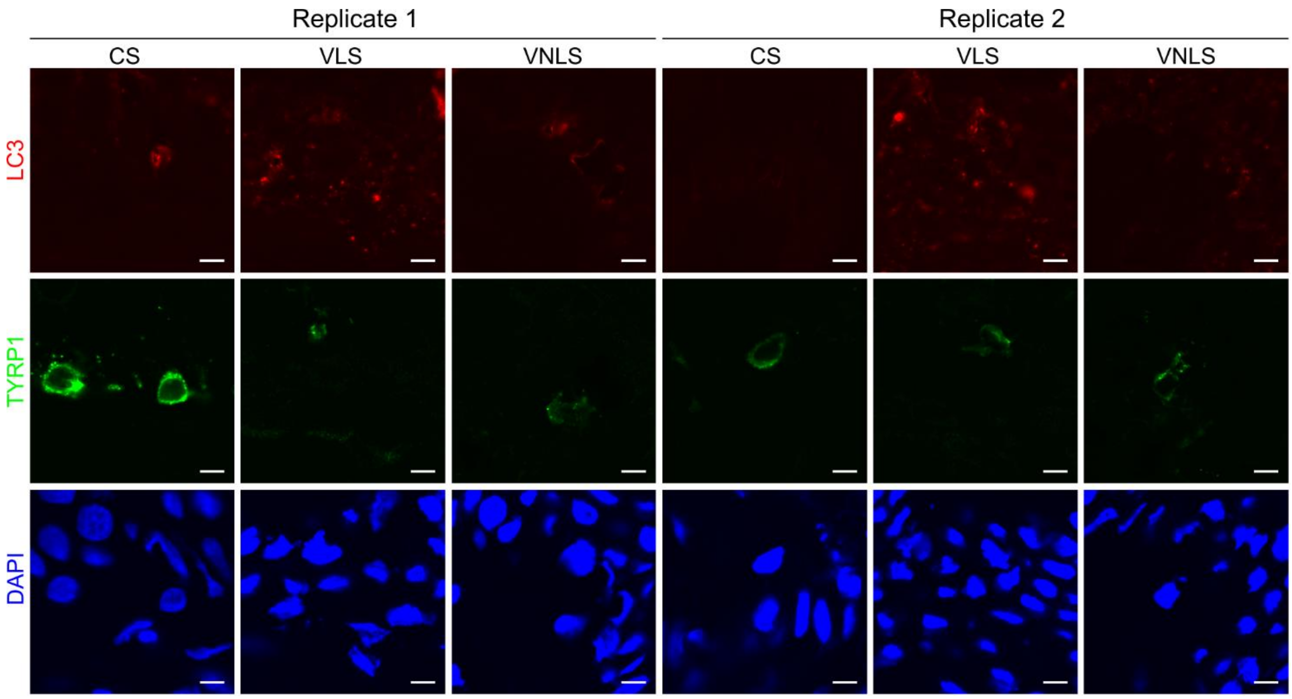

Figure S4. Split channels view of the images on Figure 5D

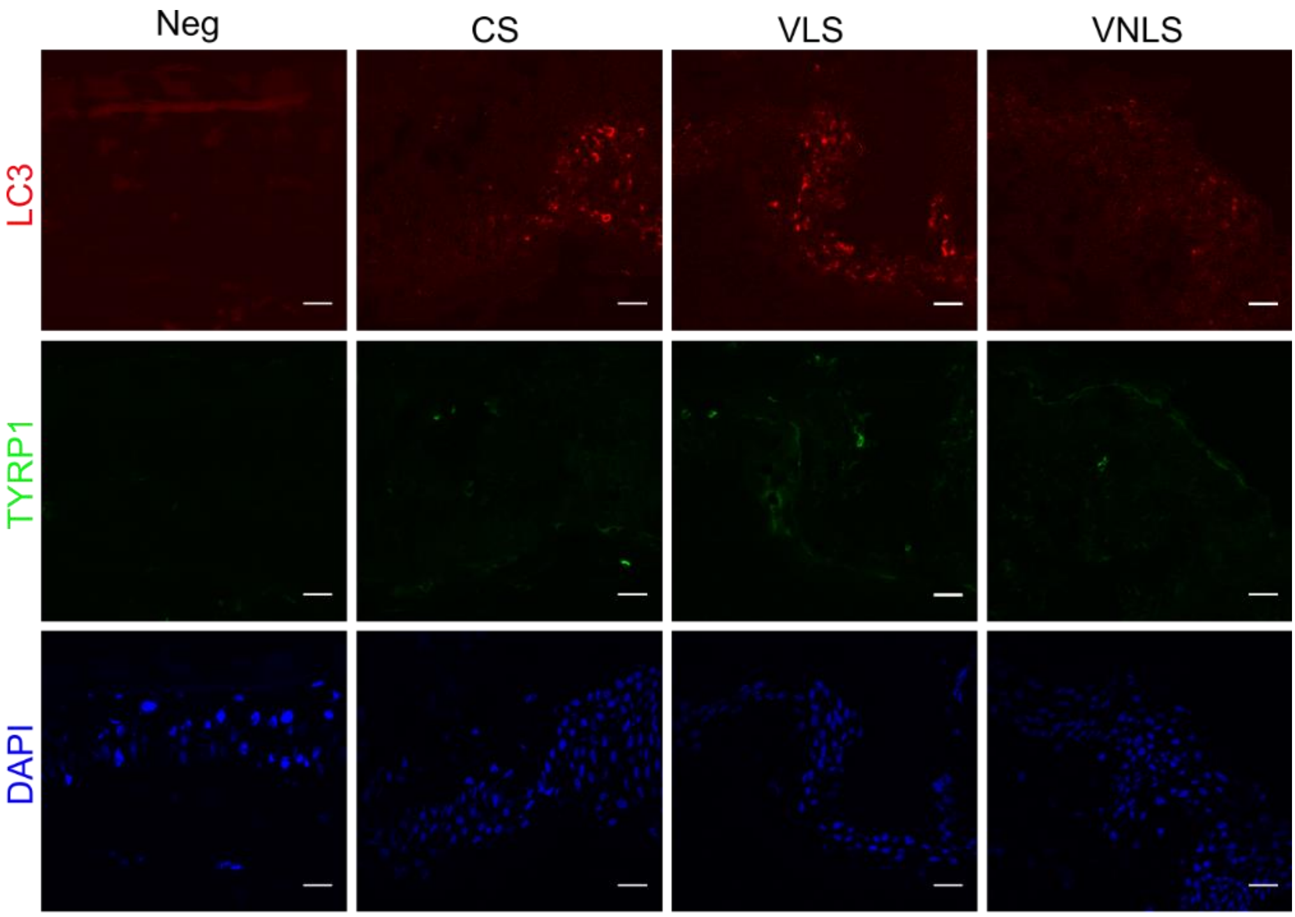

Supplement: Supplementary file 1 [file Data_Sheet_1.PDF]
